# FBXO2 modulates STAT3 signaling to regulate proliferation and tumorigenicity of osteosarcoma cells

**DOI:** 10.1186/s12935-020-01326-4

**Published:** 2020-06-16

**Authors:** Xunming Zhao, Weichun Guo, Lixue Zou, Biao Hu

**Affiliations:** Department of Orthopedics, Renmin Hospital of Wuhan University, Wuhan, Hubei China

**Keywords:** Osteosarcoma, FBXO2, STAT3, IL-6R, Degradation

## Abstract

**Background:**

Osteosarcoma (OS) is the most common primary bone malignancy in children and adolescents, and hyperproliferation of cells is a major problem of OS. FBXO2 belongs to the family of F-box proteins, and is a substrate recognition component of the Skp1-Cul1-F-box protein (SCF) E3 ubiquitin ligase complex with specificity for high-mannose glycoproteins. The aim of the present study was to investigate the critical role of FBXO2 in OS cells.

**Methods:**

The protein and mRNA expression levels of FBXO2 in clinic OS patients were measured by quantitative real time-polymerase chain reaction (qRT-PCR), Western blot and Immunohistochemical (IHC) staining assays, respectively. The FBXO2 overexpression model was constructed by retro-virus transfection in OS cells. FBXO2 knockout (KO) cells were generated by Clustered regularly interspaced short palindromic repeat (CRISPR)–CRISPR-associated protein 9 (Cas9) assay. Cell counting and colony formation assays were used to analyze the effect of FBXO2 on the biological function of OS cells. FBXO2 KO cells were injected into nude mice to observe tumor growth in vivo. The interaction between FBXO2 and IL-6 was detected by immunoprecipitation. Luciferase assay was used to determine the transcriptional activity of STAT3.

**Results:**

Here, we show that FBXO2 is significantly up-regulated in clinical OS samples compared to adjacent normal tissues. Ectopic expression of FBXO2 leads to increased OS cell proliferation and colony-forming ability, while FBXO2 knockout by CRISPR-Cas9-based gene editing has the opposite effect. In addition, the glycoprotein recognition activity of FBXO2 is required for its biological function in OS. In vivo experiments showed that FBXO2 knockout greatly impaired the tumorigenicity of OS cells in nude mice. At the molecular level, we found that knocking out FBXO2 can significantly inhibit STAT3 phosphorylation and downstream target gene expression through IL-6R stabilization.

**Conclusion:**

Together, these results indicate that FBXO2 promotes OS development by activating the STAT3 signaling pathway, suggesting that FBXO2 may be a new target for OS treatment.

## Background

Osteosarcoma (OS) is the most common primary bone malignancies in children and adolescents worldwide, representing a notably aggressive disease [1]. OS most frequently occurs in patients between 5 years and early adulthood with an increasing frequency by 0.3% per year over the last decade [2]. Despite the advance of diagnosis and detecting technology, few improvements have been made for OS treatment during the past 20 years [3]. With the advancement of targeted therapeutics, understanding of the molecular events associated with OS should be of great importance and interests to improve OS treatment [4].

Ubiquitination is catalyzed by a three steps enzymatic E1–E2–E3 cascade, participating in almost all biological processes [5]. The spatial and temporal protein ubiquitination and degradation is dominated by means of ubiquitin E3 ligase [6]. Ubiquitin E3 ligases are mainly divided into three major families: homologous to E6-associated protein C-terminus (HECT), really interesting new gene (RING), and RING-in-between-RING (RBR) E3 ligases [7]. SCF (Skp1-Cullin1-F-box) complex consists of 4 subunits, including Cullin1, Rbx1, SKP1 and an F-box protein. Cullin1 is a scaffold protein which interacts with Skp1, and also binds to Rbx1, which recruits an E2 enzyme to complete ubiquitin transferring. In this E3 complex, F-box proteins provide the substrates specificity [8].

FBXO2 protein contains an F-box associated region (FBA) domain, which is required for glycoprotein recognizing, as either deletion or mutation of this domain abolished its glycoprotein-recognizing ability [9]. FBXO2 recognizes and interacts with the innermost portion of the carbohydrate moieties of glycoproteins and plays critical roles in the regulation of both normal and abnormal neuron functions by targeting several vital neuron glycoproteins for ubiquitin mediated degradation [10]. A recent study also showed that FBXO2 targets insulin receptor (IR) for ubiquitin-dependent degradation, thereby regulating the integrity of insulin signaling [11]. However, whether FBXO2 plays a role in tumorigenesis, especially in OS, is completely unknown.

Accumulated evidences suggest that aberrant Stat3 signaling is required for the initiation, development and progression of several human cancers, including OS [12]. Cancer cells with elevated levels of activated STAT3 signaling are associated with a poor prognosis. STAT3 signaling also plays critical role in inflammatory cell-mediated transformation and tumor progression [13]. Therefore, inhibiting the STAT3 signaling is a promising therapeutic way for developing anti-cancer drugs.

The present study shows that FBXO2 regulates the proliferation of OS cells. Compared with adjacent normal tissues, FBXO2 is significantly upregulated in OS tissues. Inhibition of FBXO2 significantly impaired the activating of the canonical IL-6/STAT3 signaling pathway, which contributed to its pro-proliferation activity in OS.

## Materials and methods

### Samples, cell lines, cell culture

A total of 30 paired primary OS and adjacent non-tumor normal tissues were collected from patients (male, 13 and female, 17; age range, 8–25 years; mean age, 15.3 ± 3.8 years) with newly diagnosed with OS who had not received any previous treatment from May 2014 to Oct 2018. The present study was approved by the institutional review board of Renmin Hospital of Wuhan University and written consent was obtained from all participants or their families. Normal hFOB1.19 cells and all OS cell lines (MG63, U2OS, Saos2 and Hos) were obtained from The Cell Bank of Type Culture Collection of Chinese Academy of Sciences (Shanghai, China). Cells were cultured in RPMI 1640 supplemented with 10% fetal bovine serum (Hyclone), 100 IU/ml penicillin and 100 mg/ml streptomycin and maintained at 37 °C in a humidified atmosphere containing 5% CO_2_.

### IHC (immunohistochemical)

The protein expression levels of FBXO2 were analyzed by IHC with the corresponding anti-FBXO2 polyclonal antibody. The stained slides were scored by two investigators according to the value of IRS systems.

### Transfection

All plasmids were purchased from Shanghai GeneChem Co., Ltd. (Shanghai, China). For transfection, U2OS cells were cultured as described to 70–80% confluence in a 6-well plate. Plasmids were co-transfected using Lipofectamine2000 (Thermo Fisher Scientific, Inc.) following the manufacturer’s instructions.

### CRISPR/Cas9 knock out (KO) cell lines

The FBXO2 knock-out OS cells were generated by CRISPR/Cas9 technology based on the PX459 vector. Primers used for FBXO2 were list: Forward primer: CACCGACCTTCTGCGTAACCCGTGT). Reverse primer: AAACACACGGGTTACGCAGAAGGTC). 1.5 × 10^5^–2.5 × 10^5^ U2OS or MG63 cells were seeded in a 6-well tissue culture plate in 3 ml of antibiotic-free standard growth medium per well. Cells about 40–60% confluency were ready for transfection. Cells were transfected with PX459-FBXO2 plasmid using Lipofectamine 2000 (Thermo Fisher Scientific, Inc.) following the manufacturer’s instructions. 24 h later, cells were cultured into RPMI 1640 supplemented with 10% fetal bovine serum. Then cells were selected with 2 µg/ml puromycin for about 2 weeks. Different single clones were then isolated and expanded. The knock out efficiency was verified by western blot with anti-FBXO2 antibody.

### Quantitative real time-polymerase chain reaction (qRT-PCR)

Total RNA was extracted from human tissue samples or cells by using the RNA Isolation kit (Ambion, Thermo Fisher Scientific, Inc.) according to the manufacturer’s instructions. 1 µg RNA was used to generate cDNA. First-strand cDNA synthesis was obtained using the Reverse Transcription System (Promega). Oligo dT was used to prime cDNA synthesis. qPCR was performed using a SYBR Green Premix Ex Taq (Takara Bio, Inc., Otsu, Japan) on Light Cycler480 (Roche, Basel, Switzerland). cDNA was diluted for 20 times for qRT-PCR assay. PCR conditions included an initial holding period at 95 °C for 5 min, followed by a two-step PCR program consisting of 95 °C for 5 s and 60 °C for 30 s for 50 cycles, and 72 °C for 5 min. Differences in gene expression, expressed as fold-changes, were calculated using the 2^−ΔΔCq^ method. Results were normalized to β-actin. The primer sequences used are available upon request.

### Immunoprecipitation (IP)

Cells transfected with indicated plasmids were lysed in lysis buffer (150 mM Tris–HCl pH 7.5, 150 mM NaCl, 0.5% Nonidet P40, and 50 mM PMSF) for 30 min at 4 °C. Lysates were cleared using centrifugation, the supernatant was then subjected to IP with 20 μl Flag M2 beads (Sigma) overnight at 4 °C with gentle rotation. Beads were washed with lysis buffer for 6 times and precipitates were denatured in 2X SDS buffer at 95 °C for 5 min.

### Cycloheximide (CHX) assay

To analyze protein half-life, cells were treated with CHX (20 μg/ml) for different durations followed by western blot assay.

### Western blotting

Tissues and cells were lysed with 2× SDS lysis buffer. Proteins were separated by 10% SDS-PAGE, transferred to nitrocellulose (NC) membranes, incubated with primary and secondary antibodies. The following primary antibodies were purchased: mouse anti-human FBXO2 (1:500, cat. no. sc-398111, Santa Cruz Biotechnology, Inc., Santa Cruz, CA, USA), mouse anti-ubiquitin (1:1000, cat. no. sc-166553, Santa Cruz Biotechnology, Inc.), mouse anti-IL-6Rα (1:500, cat. no. sc-373708, Santa Cruz Biotechnology, Inc.), mouse anti-gp130 (IL6ST) (1:500, cat. no. sc-376280, Santa Cruz Biotechnology, Inc.), mouse anti-Ki-67 (1:500, cat. no. sc-23900, Santa Cruz Biotechnology, Inc.), mouse anti-Flag M2 (1:5000, cat. no. F1804, Sigma-Aldrich, USA), mouse anti-HA (1:5000, cat. no. H9658, Sigma-Aldrich, USA), rabbit anti-human Phospho-Stat3 (Tyr705) (D3A7) (1:1000, cat. no. 9145S, Cell Signaling Technology, USA), mouse anti-human Stat3 (124H6) (1:1000, cat. no. 9139, Cell Signaling Technology, USA), mouse anti-human actin (1:10,000, cat. no. sc-8432, Santa Cruz Biotechnology, Inc.).

### Luciferase reporter assays

The HIF-1alpha-, STAT1- or STAT3-Luc plasmids were generated by insert synthetic response elements recognized by these transcriptional factors into pGL4.15 vector (Promega, Madison, Wisconsin, USA). For the luciferase reporter assays, U2OS cells were co-transfected HIF-1alpha-, STAT1- or STAT3-Luc with shRNA-FBXO2 or shRNA-NC using Lipofectamine 2000 (Invitrogen, USA). Cells were harvested 36 h after transfection, and luciferase activity was measured using the dual Luciferase reporter assay system (Promega).

### Chromatin immunoprecipitation (Chip)

We used a chromatin immunoprecipitation assay kit (Millipore, USA). In brief, cells were fixed with 1% formaldehyde (Beyotime Biotechnology, China) and harvested in SDS lysis buffer. DNA was sheared to fragments about 200–1000 bp by repeated sonication. Lysates containing soluble chromatin were precipitated overnight with 2 μg of anti-Stat3 antibody (124H6) (Cell Signaling Technology, USA) antibody or mouse IgG (#ab172730, Abcam). Protein A/G agarose was then added for additional 4 h. Protein-DNA crosslinks were reversed by treatment with proteinase K for 2 h at 45 °C. The DNA was subsequently purified, diluted and subjected in the quantitative real-time PCR reactions. The human XIAP and MCL1 gene promotor fragments were amplified. The promotor region of human GAPDH was used as a negative control. The primer sequences used are available upon request.

### GST pull-down assay

GST or GST-FBXO2 proteins were expressed in BL21 cells and purified using glutathione-Sepharose beads (Beyotime Biotechnology, China) in binding buffer (100 mM NaCl, 50 mM Tris–HCl pH 7.5, 1 mM dithiothreitol, 2 mg/ml leupeptin, 2 mg/ml aprotinin, and 100 mg/ml phenylmethylsulfonyl fluoride). Equal amounts of GST or GST-FBXO2 proteins were resuspended in reaction buffer (200 mM NaCl, 50 mM HEPES pH 7.5, 1 mM MgCl2, and 0.2% Triton X-100) containing 0.2 mg/ml BSA and incubated for 2 h. Then, 1 mg Flag-IL6ST or Flag-IL6Ra-transfected cell lysate were added to each mixture followed by rotation for 1 h. The beads were boiled in SDS loading buffer and the eluted proteins were subjected to immunoblotting analysis.

### In vivo xenograft study

BALB/c nude mice were randomly divided into two groups with 5 in each group. FBXO2 WT or KO U2OS cells (1 × 10^7^ cells) were suspended in HBSS/Matrigel mix (1:1 volume) and injected subcutaneously in the left flank of nude mice at 0.2 ml/mice for about 6 weeks. Alternatively, FBXO2 WT U2OS cells were injected subcutaneously in the right flank of nude mice, and FBXO2 KO U2OS cells were injected subcutaneously in the left flank of the same mice at 0.2 ml/mice for about 6 weeks. Tumor volumes were determined using a caliper. The results were converted to tumor volume (mm^3^) by the formula length × width^2^ × 1/2. Tumor weights were measured at the day mice were sacrificed.

### Statistical analysis

Data are presented as the mean ± standard deviation of three independent experiments. Statistical significance was evaluated using student t-test. Data were analyzed using GraphPad PRISM 6 (GraphPad Software, Inc., La Jolla, CA, USA) and Statistical significance is displayed as *p < 0.05, **p < 0.01 or ***p < 0.001.

## Results

### FBXO2 is overexpressed in OS samples and cell lines

**Fig. 1:**
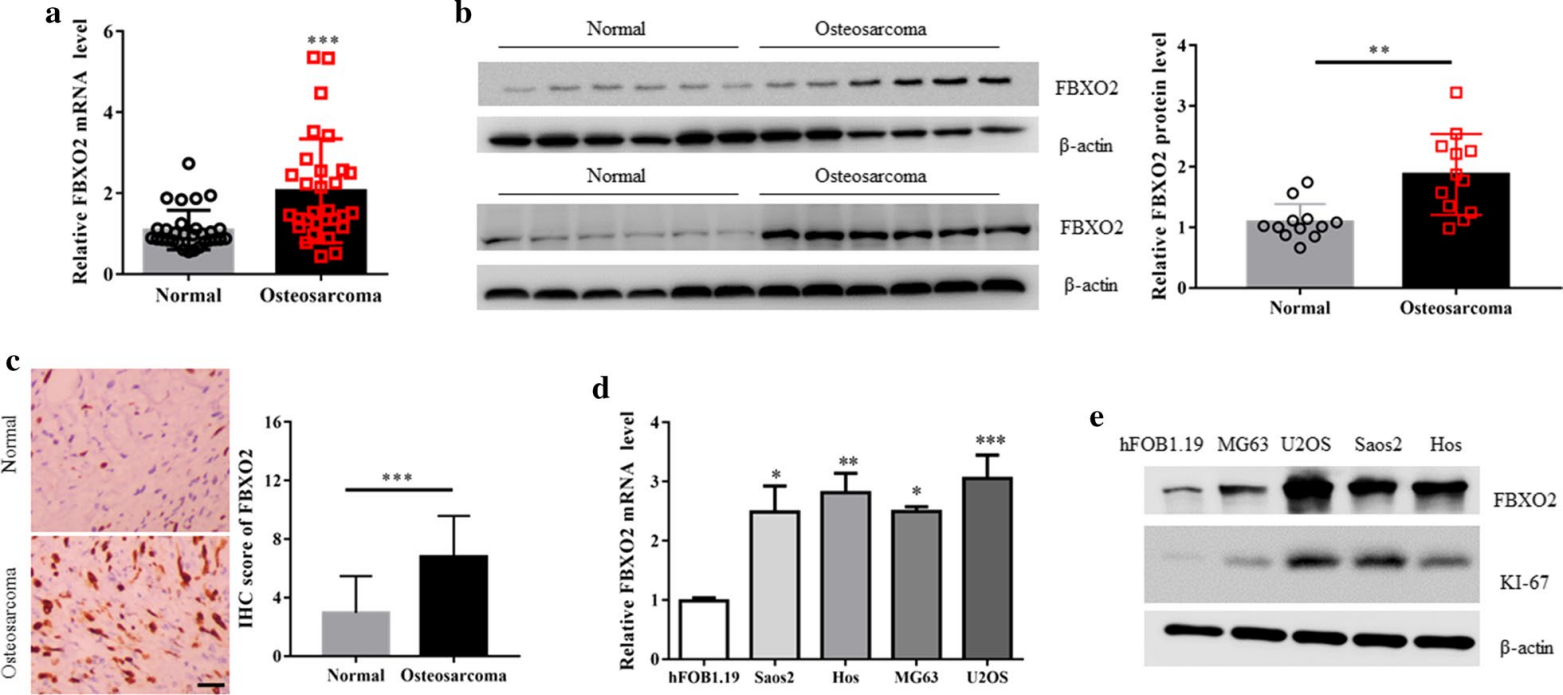
FBXO2 is upregulated in OS samples and cell lines. **a** FBXO2 mRNA and **b** protein expression in OS and adjacent normal tissues. The quantification plot was based on scanning densitometry analysis using the Image J software (v 1.8.0). **c** Immunohistochemical analyses of 20 OS and adjacent normal tissues using anti-FBXO2 antibody were performed. Representative images of IHC staining were presented. The scale bar represents 50 μm. The quantitative analysis for IHC images were shown. **d** FBXO2 mRNA and protein **e** expression in four OS cell lines and normal human osteoblastic hFOB1.19 cells

To investigate the expression of FBXO2 in clinic OS samples, we first measured the mRNA levels of FBXO2 in 30 paired primary OS and adjacent normal tissues by qRT-PCR. Compared with adjacent normal tissues, FBXO2 was notably increased in OS samples (Fig. 1a). In concert with this, FBXO2 proteins expression were also significantly upregulated in 12 OS samples compared to adjacent normal ones by western blotting and immunohistochemistry (IHC) assay using anti-FBOX2 antibody (Fig. 1b, c). Compared with normal osteoblast hFOB1.19 cells, the mRNA and protein expression levels of FBXO2 were also upregulated in all OS cell lines we chose (Fig. 1d and e). Interestingly, the overexpression of FBXO2 is associated with high levels of KI-67 protein in these OS cells (Fig. 1e), suggesting that FBXO2 may play a role in OS cells proliferation. Taken together, these data indicate that FBXO2 is overexpressed in OS samples and cell lines.

### FBXO2 regulates the OS cells proliferation in vitro and in vivo

**Fig. 2:**
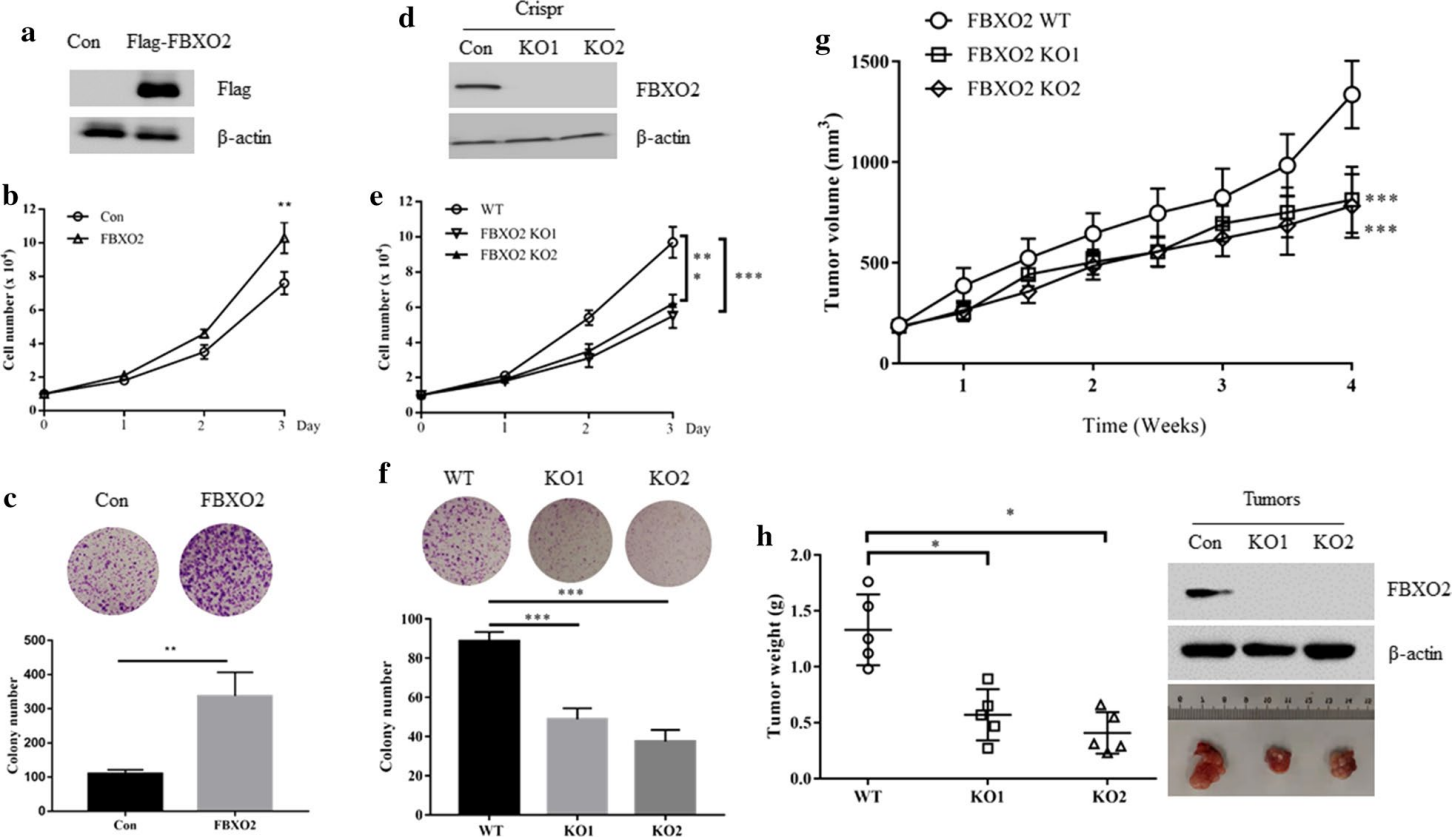
FBXO2 regulates the OS cells proliferation in vitro and in vivo. **a** FBXO2 protein expression in MG63 cells with or without Flag-FBXO2 overexpression. **b** Cell growth curve of MG63 cells with or without Flag-FBXO2 overexpression. **c** Colony formation assay of MG63 cells with or without Flag-FBXO2 overexpression. **d** FBXO2 KO U2OS cells were generated by CRISPR assay and detected by western blot. **e** Cell growth curve of FBXO2 WT or FBXO2 KO U2OS cells. **f** Colony formation assay of FBXO2 WT or FBXO2 KO U2OS cells. **g** 1 × 10^7^ FBXO2 WT or KO U2OS cells were injected subcutaneously into BALB/c nude mice, tumour growth was measured using a caliper at the indicated times after injection. n = 5 for each group. **h** Mice were sacrificed 6 weeks after transplantation. The tumors were then excised and weighed and subjected to western blot assay with indicated antibodies

To investigate the biological function of FBXO2 in OS cells, we chose MG63 cells to overexpress Flag-FBXO2 because it contains relatively low FBXO2 protein expression levels (Fig. 2a). Ectopic expression of FBXO2 significantly promoted the proliferation and colony formation of MG63 cells (Fig. 2b, c). Because U2OS cells have the relatively highest FBXO2 expression level, the CRISPR/Cas9 system was used to generate the FBXO2 KO OS cell line. Single clones were then selected and verified by Western blot analysis using anti-FBOX2 antibody (Fig. 2d). Compared with control cells, FBXO2 KO cells showed reduced cell proliferation (Fig. 2e). Consistent with this observation, FBXO2 depletion was accompanied by a decrease in colony-forming ability in OS cells (Fig. 2f). Similar phenotypes were also observed in FBXO2 overexpressed U2OS and FBXO2 depleted MG63, indicating that FBXO2 regulated OS cell proliferation in vitro (Additional file 1: Fig. S1A–F). To investigate the biological function of FBXO2 in vivo, BALB/c nude mice were randomly divided into two groups, 5 in each group. FBXO2 WT or KO U2OS cells were injected subcutaneously into nude mice. We observed that knockout of FBXO2 significantly inhibited tumor growth, as depicted by measuring tumour volumes and weights (Fig. 2g, h, Additional file 1: Fig. S1G).

### FBXO2 activates STAT3 signaling in OS dependent on its glycoprotein recognizing activity

**Fig. 3:**
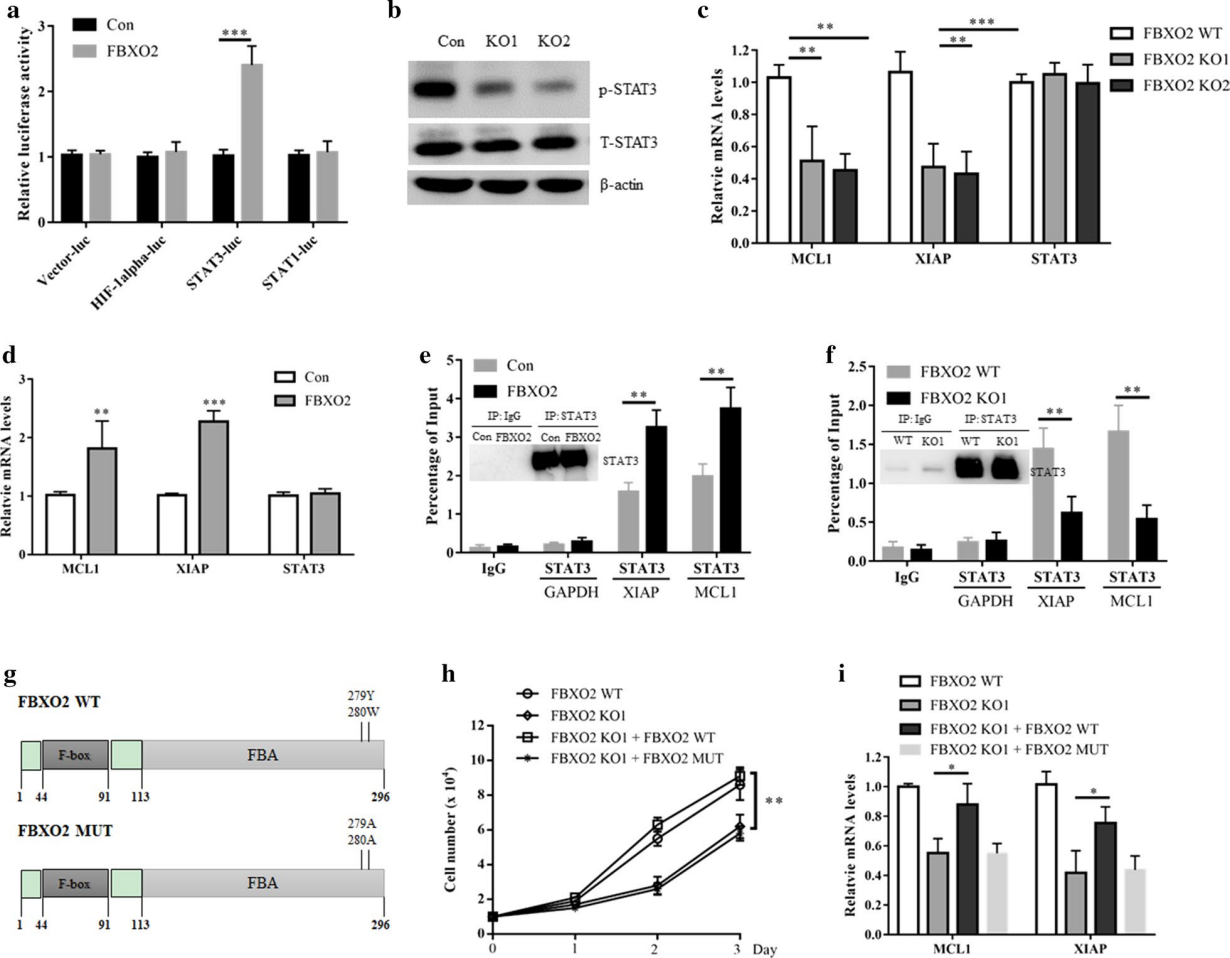
FBXO2 activates STAT3 signaling in OS dependent on its glycoprotein recognizing activity. **a** U2OS cells were transfected with the indicated plasmids for 36 h and then cells were lysed for luciferase assay. The results are indicated as fold induction of luciferase activity from triplicate experiments. ***p < 0.001. **b** U2OS cells were transfected with FBXO2 plasmid for 36 h, cells were lysed for western blot analysis. **c** The mRNA levels of MCL1, XIAP and STAT3 in FBXO2 WT or KO U2OS cells. **d** The mRNA levels of MCL1, XIAP and STAT3 in U2OS cells transfected with or without FBXO2. **e** ChIP assay showed that overexpression of FBXO2 increased the binding of STAT3 to the promotors of both MCL1 and XIAP genes in U2OS cells. The enrichment efficiency of endogenous STAT3 protein was detected by western blot assay. **f** ChIP assay showed that in FBXO2 KO U2OS cells, the binding of STAT3 to the promotors of both MCL1 and XIAP genes was decreased. The enrichment efficiency of endogenous STAT3 protein was detected by western blot. **g** The diagram of FBXO2 WT and FBXO2 MUT. FBXO2 WT or FBXO2 MUT plasmids were transfected into U2OS FBXO2 KO cells for 36 h. **h** The cell growth curve and **i** the mRNA levels of MCL1 and XIAP in these cells

To investigate which downstream signaling pathway is regulated by FBXO2, we co-transfected FBXO2 with several luciferase reporter gene plasmids (including HIF-1alpha-, STAT1- or STAT3-derived luciferase reporter genes) into U2OS cells. We found that FBXO2 specifically activated STAT3-derived luciferase activity, indicating that STAT3 signaling may be a downstream signaling pathway regulated by FBXO2 (Fig. 3a). In addition, knockout of FBXO2 decreased the phosphorylation of STAT3 in U2OS cells, without affecting the total STAT3 protein (Fig. 3b). Moreover, the mRNA levels of STAT3 target genes MCL1 and XIAP [14], but not STAT3 itself, were decreased in FBXO2 KO cells and induced in FBXO2 overexpressed cells (Fig. 3c, d). Importantly, in a Chip assay, we found that overexpression of FBXO2 increased, while knockout of FBXO2 reduced the STAT3 binding at both XIAP and MCL1 promotors (Fig. 3e, f). Taken together, these results indicate that FBXO2 plays a regulatory role in STAT3 signaling. FBXO2 is a promiscuous ubiquitin ligase with many partners, especially glycoproteins [15]. Importantly, according to the previous report, the F-box–associated (FBA) domain of FBOX2 is required for its glycoprotein recognizing, which could be completely abolished by mutations of two residues [16] (Fig. 3g). To clarify whether glycoprotein recognizing activity is required for the oncogenic function of FBXO2 in OS, we restored the expression of FBXO2 in FBXO2 KO cells. Interestingly, restored the expression of the wild type (WT), but not the F-box–associated domain mutant (MUT) FBXO2 in FBXO2 KO cells, promoted OS cells re-proliferation and STAT3 downstream genes expression (Fig. 3h, i). Thus, these data implied that FBXO2 promoted OS proliferation and STAT3 signaling activation dependent on its glycoprotein recognizing activity.

### FBXO2 interacts with interleukin 6 receptor to stabilize it

**Fig. 4:**
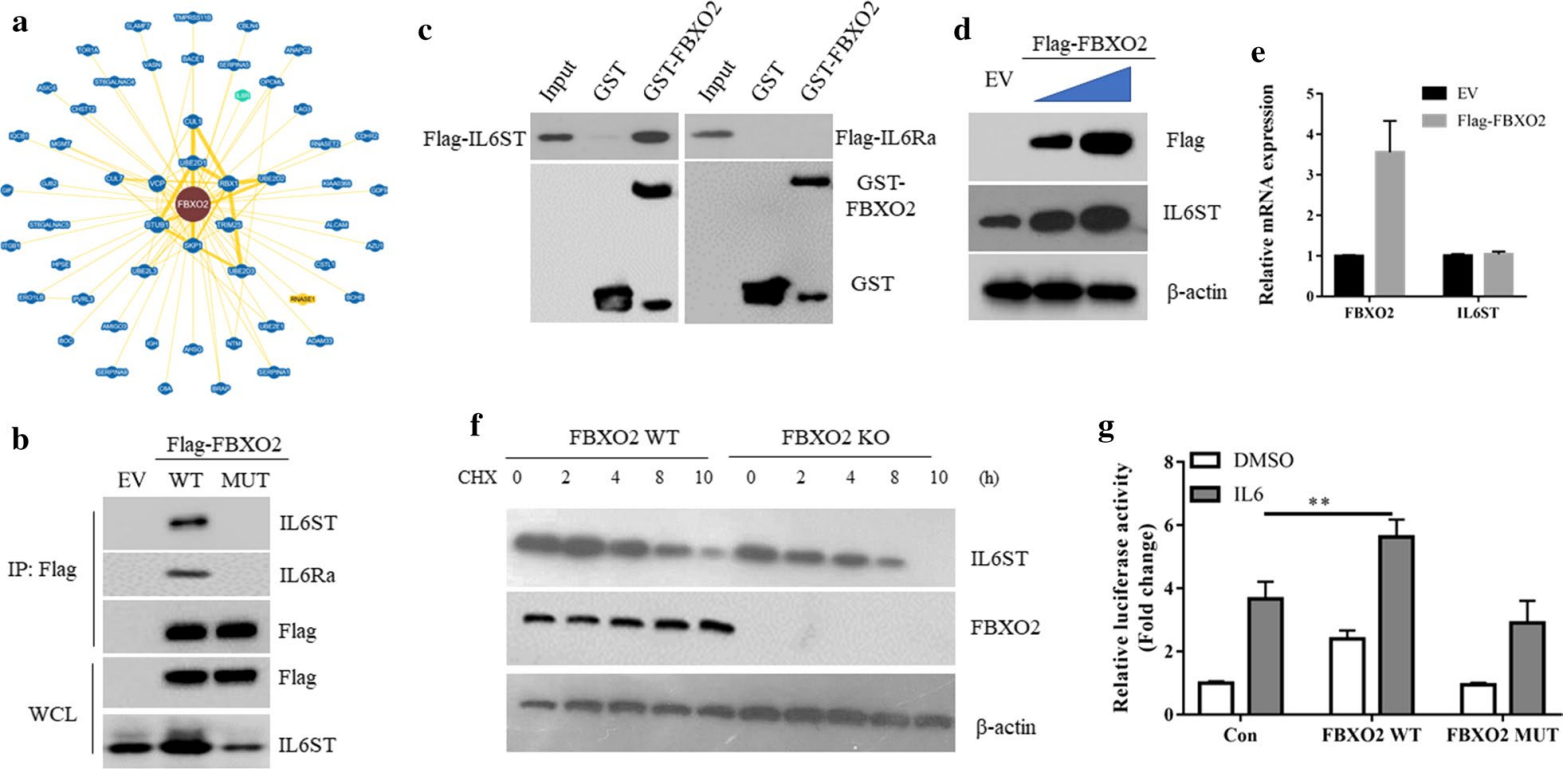
FBXO2 interacts with interleukin 6 receptor to stabilize it. **a** The interaction protein network of FBXO2 revealed by the BioGRID database. **b** 293T cell were transfected with EV, Flag-FBXO2 WT or MUT for 36 h. Cells were lysed and immunoprecipitated with Flag M2 beads. The immunoprecipitates were detected by western blot using indicated antibodies. **c** Beads coated with bacterially expressed GST or GST-SENP2 were incubated with purified Flag-IL6ST or Flag-IL6Ra protein. Beads were washed, and the bound proteins were analyzed by Western blotting with indicated antibodies. **d** Immunoblotting analysis of the cell lysates of MG63 cells transfected with increased doses of FBXO2WT. **e** qRT-PCR analysis of IL6ST and FBXO2 in MG63 cells transfected with EV or Flag-FBXO2 WT. **f** Cell lysates from FBXO2 WT and KO U2OS cells treated with 20 μg/ml cycloheximide (CHX) were subjected to western blot with the indicated antibodies. **g** U2OS cells were co-transfected STAT3-Luc with indicated plasmids. 24 h after transfection, cells were treated with DMSO or IL-6 (5 ng/ml) for additional 12 h. The cells were lysed and assayed for luciferase assay as above described. **p < 0.01

To further understand how FBXO2 regulated the STAT3 signaling, we searched the interacting proteins of FBXO2 through the BioGRID database (https://thebiogrid.org/). A total of 54 proteins have been shown to be associated with FBXO2, including the known SKP1 and Cullin1. Interestingly, we found that Interleukin 6 receptor (IL-6R), a key glycoprotein in mediating IL-6-STAT3 signaling [17], is listed as an interacting protein of FBXO2 by high throughput assay (Fig. 4a). IL-6R is a receptor complex consisting of IL6Ra and IL6ST (interleukin 6 signal transducer (IL6ST/GP130/IL6-beta). The interaction between FBXO2 and IL-6R was confirmed by the co-immunoprecipitation assay, as both IL6Ra and IL6ST proteins were detected in the anti-Flag-FBXO2WT immunoprecipitated protein complex (Fig. 4b). To further show which protein is directly interacted with FBXO2, the GST-pull assays were used. We found that IL6ST but not IL6Ra was associated with GST-FBXO2 in vitro (Fig. 4c), suggesting IL6ST might bridge the interaction between FBXO2 and IL6Ra in vivo. However, instead of degradation, overexpression of FBXO2WT increased the protein expression of IL6ST (Fig. 4d), without affecting its mRNA level (Fig. 4e). Moreover, in the absence of FBXO2, the half-life of IL6ST was decreased (Fig. 4f). Importantly, FBXO2 MUT failed to bind to IL-6R and overexpression of FBXO2 MUT also failed to induce IL6ST expression (Fig. 4b), suggesting IL6ST is the glycoprotein which mediated the function of FBXO2 in the regulation of STAT3 signaling. In agreement, overexpression of FBXO2WT, but not FBXO2MUT, enhanced IL-6-induced STAT3 activation (Fig. 4g).

## Discussion

Initially, FBXO2 was found to be specifically expressed in the nervous system and FBXO2 KO mice developed age-related cochlear degeneration [9, 18]. A recent study has found that FBXO2 is highly expressed in gastric cancer and the overall survival (OS) of patients with high FBXO2 expression is significantly shorter than patients with low FBXO2 expression [19]. FBXO2 regulated the epithelial-mesenchymal transition (EMT) in human gastric cancer cells, suggesting FBXO2 plays an oncogene role in gastric cancer. However, it is unclear whether FBXO2 has a role in other tumors.

In the present study, we demonstrated that FBXO2 exerted oncogenic effects towards OS cells, which were supported by several lines of evidences. Firstly, both the mRNA and protein levels of FBXO2 were overexpressed in OS tissue samples and cultured cell lines, respectively. Secondly, FBXO2 overexpression increased the proliferation and colony formation ability of OS cells, whereas knockout of FBXO2 inhibited OS cells proliferation in vitro and tumorigenicity in vivo. Thirdly, overexpression of FBXO2 activated STAT3 signaling in OS, whereas knockout of FBXO2 inactivated it. We further demonstrated that the glycoprotein recognizing ability of FBXO2 was required for its biological function. We showed that FBXO2 used its FBA domain to bind to IL-6R and overexpression of FBXO2 stabilized IL-6R. Persistent activation of the IL-6 signaling pathway is detrimental to the bone tissue and might ultimately result in the development of OS [20]. Ligation of IL-6 to IL-6R activates the Janus protein tyrosine kinase (JAK) to phosphorylate and activate STAT3 [12]. Our data showed that overexpression of FBXO2WT, but not FBXO2MUT, enhanced IL-6-induced STAT3 activation, indicating FBXO2-mediated IL-6R stabilization is required for STAT3 activation. FBXO2 has been reported to bind to APC2 and increased total APC2 levels, suggesting a novel role of FBXO2 independent from its function as ubiquitin ligase [21]. Although the detailed mechanisms underlined the stabilization of IL-6R by FBXO2 were currently unclear, our data showed a critical role of FBXO2 in the regulation of the STAT3 signaling and OS progress via IL-6R stabilization.

In summary, our gain- and loss-of-function studies demonstrate that FBXO2 plays an important role in OS cells proliferation via modulating the STAT3 signaling pathway. Our results indicate that FBXO2 dysregulation is a critical feature of OS. In many tumors, IL-6 and its receptor IL-6R are highly expressed, and the high expression of IL-6 is closely related to the poor clinical prognosis of tumor patients [22]. At present, biotherapies targeting the IL-6/STAT3 signaling pathway are mainly focused on monoclonal antibodies such as IL-6 and IL-6R. Among them, anti-IL-6 monoclonal antibody (Siltuximab) has been used in clinical trials to treat cancers [23, 24]. In the present study, we found that FBXO2 can affect the stability of IL-6R, and knocking out FBXO2 can inhibit the proliferation of osteosarcoma cells both in vitro and in vivo. Therefore, inhibiting the expression or activity of FBXO2 may be a promising therapeutic strategy for the treatment of OS patients. However, due to the relatively small number of clinical samples we use, future studies involving larger clinical samples and FBXO2 gene knockout mice should be conducted to further clarify the biological function of FBXO2 in the development and treatment of OS.

## Conclusion

Our study showed a critical role of FBXO2 in OS cells proliferation and tumorigenesis. Our findings provide a novel molecular mechanism for the positive regulation of the STAT3 signaling by FBXO2 in OS. The stabilization of IL-6R by FBXO2 is dependent on its FBA domain, but independent of its ubiquitin ligase activity. The interference of this regulation of FBXO2 significantly inhibited the proliferation of OS cells both in vitro and in vivo. Therefore, our data indicate that FBXO2 is a potential therapeutic target in OS, and inhibition of FBXO2 should be a prospective strategy for OS treatment.

## Supplementary information

**Additional file 1: Figure S1**. FBXO2 regulates the OS cells proliferation in vitro and in vivo.

## Data Availability

Please contact corresponding author for data requests.

## Abbreviations

OS: Osteosarcoma
qRT-PCR: Real time-polymerase chain reaction
SCF: Skp1-Cul1-F-box protein
HECT: Homologous to E6-associated protein C-terminus
RING: Really interesting new gene
RBR: RING-in-between-RING
FBA: F-box associated region
IP: Immunoprecipitation
CHX: Cycloheximide
