# Supplementary material for: FBXO2 modulates STAT3 signaling to regulate proliferation and tumorigenicity of osteosarcoma cells
Source: Cancer Cell Int. 2020 Jun 16;20:245. doi: 10.1186/s12935-020-01326-4 (PMC7296666; doi:10.1186/s12935-020-01326-4)

Additional file Figure legends

Figure S1 FBXO2 regulates the OS cells proliferation in vitro and in vivo.

Relative to Figure 2

(A) FBXO2 protein expression in U2OS cells with or without Flag-FBXO2 overexpression. (B) Cell growth curve of U2OS cells with or without Flag-FBXO2 overexpression. (C) Colony formation assay of U2OS cells with or without Flag-FBXO2 overexpression. (D)FBXO2 KO MG63 cells were generated by CRISPR assay and detected by western blot. (E) Cell growth curve of control or FBXO2 KO MG63 cells. (F) Colony formation assay of control or FBXO2 KO MG63 cells. (G) FBXO2 WT U2OS cells were injected subcutaneously in the right flank of BALB/c nude mice, and FBXO2 KO U2OS cells were injected subcutaneously in the left flank of the same mice at 0.2 ml/mice for about 6 weeks.

Figure S1


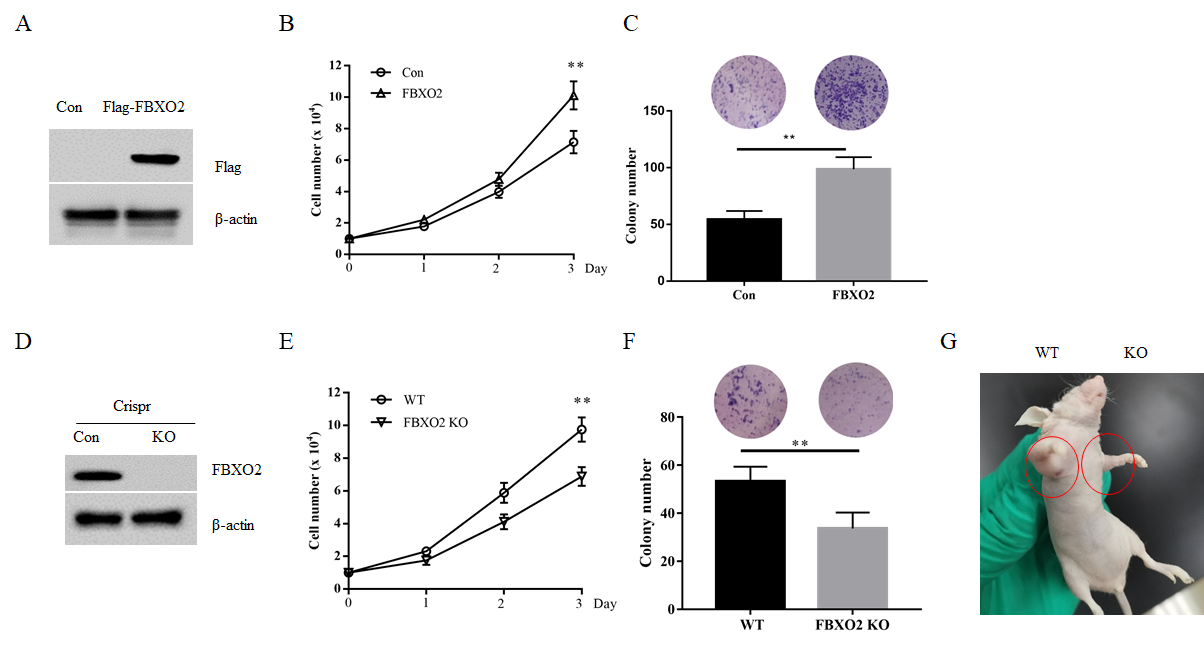

Supplement: Supplementary file 1 — Additional file 1: Figure S1. FBXO2 regulates the OS cells proliferation in vitro and in vivo. [file 12935_2020_1326_MOESM1_ESM.docx]
